# Uncertainty Determination Methodology, Sampling Maps Generation and Trend Studies with Biomass Thermogravimetric Analysis

**DOI:** 10.3390/ijms11103660

**Published:** 2010-09-28

**Authors:** Jose A. Pazó, Enrique Granada, Ángeles Saavedra, Pablo Eguía, Joaquín Collazo

**Affiliations:** 1 ETS Ingenieros Industriales, University of Vigo, Lagoas-Marcosende s/n 36200–Vigo, Spain; E-Mails: jpazo@uvigo.es (J.A.P.); peguia@uvigo.es (P.E.); 2 ETS Ingeniería de Minas, University of Vigo, Lagoas-Marcosende s/n 36200–Vigo, Spain; E-Mail: saavedra@uvigo.es

**Keywords:** solid biofuel, sampling methodology, uncertainty, prompt analysis, TG analysis

## Abstract

This paper investigates a method for the determination of the maximum sampling error and confidence intervals of thermal properties obtained from thermogravimetric analysis (TG analysis) for several lignocellulosic materials (ground olive stone, almond shell, pine pellets and oak pellets), completing previous work of the same authors. A comparison has been made between results of TG analysis and prompt analysis. Levels of uncertainty and errors were obtained, demonstrating that properties evaluated by TG analysis were representative of the overall fuel composition, and no correlation between prompt and TG analysis exists. Additionally, a study of trends and time correlations is indicated. These results are particularly interesting for biomass energy applications.

## 1. Introduction

After the Kyoto protocol [1] and the 2009 Copenhagen United Nations Climate Change Conference, environmental policies have focused on climate protection. A way to advance and accelerate the progress in this area, is to reduce the use of fossil fuels for energy production by increasing production of renewable and CO_2_-neutral energy sources such as biomass [2].

Pazó *et al.* [3] considered the study of sampling maps generation and the uncertainty determination methodology for four materials: hazelnut shell, brassica pellets, poplar pellets and pine nut shell.

In this paper, the work initiated by Pazó *et al.* [3] is extended with the study of four other types of biomass: almond shell, ground olive stone, pine pellets and oak pellets. In addition to applying the same procedure to a new set of materials, a numerical study of linear trends and time correlations is presented for all eight types of biomass.

As in the previous article, TG analyses were used to provide information concerning the chemical composition, thermal behavior and reactivity of biomass in a straightforward manner [4,5]. Many studies on the accuracy of TG experiments have been published [6–10], and various sampling methods have been proposed. Currently, TG methodologies are often based on small samples obtained from large batches. Thus, careful reduction is necessary to prevent segregation and stratification problems [9]. A good sampling method should be able to achieve a representative sample without being affected by the aforementioned problems.

A new methodology for the sampling of solid biomass and determination of error associated with the measurement of thermal properties was presented [11,12] and validated in a prompt analysis.

By using this sample method, this paper first presents the materials used in the study and the statistical method used to choose the samples. In a following section, the thermogravimetric method used and the statistical treatment of data are explained in detail. Next, the results of TG analysis for the four types of biomass are described, revealing the moisture, volatile, ash and fixed carbon content of each. Moisture content affects the heating value of biomass, and ash determines the level of fouling and corrosion [13,14]. Moreover, volatile compounds influence the behavior of the flame. These aspects reveal the intrinsic heterogeneity values, giving us the minimum sizes of the samples to a preset error or the errors made for a default sample size. Additionally, the confidence intervals and the correlations between the moisture, volatile matter and ash content of the materials are presented. The data suggest that there is no correlation between the results of different analyses.

Finally, a study of the linear trend and the random variation components for the properties of eight materials is presented. The Pearson correlation was utilized to check the presence of linear trends, and the Ljung-Box test employed to verify the correlation in time of the random variation.

This method may contribute to a wider and more correct application of biomass for energetic purposes.

## 2. Experimental Section

All materials were handled in the same laboratory by the same analyst. Because the materials were exposed to environmental conditions for less than half an hour, the effects of environmental variations in the properties of the materials were ignored (variations in temperature and relative humidity were considered insignificant over such a short period of time). Laboratory instruments were verified and calibrated to assure that the experimental methodology was accurate. Errors registered during the experiments were considered to be non-systematic errors and were related to the precision of the experiment. Thus, these errors were quantified in the total sampling error.

### 2.1. Materials

Several lignocellulosic materials derived from agricultural waste and forestry materials were investigated. Thus, the broad spectrum of solid biomass that can be used as fuel in combustion processes was evaluated. Agricultural materials, almond shell (As) and ground olive stone (Gos) were stored in large bags, while forestry oak pellets (Op) and pine pellets (Pin) were stored in sacks.

### 2.2. Sampling and Reduction of the Samples

Depending on the material, sampled masses varied from 320 × 10^−3^ kg to 730 × 10^−3^ kg. Fuel samples were obtained from a tube sampler, which was designed according to the requirements specified in CEN/TS [15] and the work of Pierre Gy [16]. The sampling methodology used to obtain the fuel samples is described in the literature [11,12], along with the method used to reduce the samples. Fuel samples were obtained through a tube sampler, which was designed to work with all kinds of solid biomass. The nominal maximum size “d” of the material sampled is taken as 20 mm [12], so the tube sampler should be able to collect at least V_min_ = 0.05 · d = 0.05 · 20 = 1 dm^3^ = 10^−3^ m^3^ [12]. Table 1 shows the average weight of samples selected for TG analysis. Tweezers were used to place the samples into the crucibles.

### 2.3. TG analysis Methodology

All experiments were performed on a TG-DTA/DSC SETARAM Labsys electronic thermobalance, which can achieve a maximum temperature of 1600 °C and heating rates from 0.001 to 50 °C·min^−1^. To avoid heat and mass transfer limitations, approximately 20 × 10^−6^ kg of sample were used, and platinum crucibles without lids were employed. All experiments were initially conducted under an inert flow of nitrogen at a rate of 45 mL·min^−1^, to prevent the samples from oxidizing and to determine the concentration of moisture and volatile material. Subsequently, dry air (45 mL·min^−1^) was used to determine the ash content. The parameters of the thermal analysis are shown in Table 2.

Steps 1 through 4 were conducted to determine the moisture content, while steps 5 through 10 were performed to determine the concentration of volatile material. Lastly, steps 11 through 13 were conducted to determine the ash content of the biomaterials. Most of the steps were not directly related to the determination of moisture, volatile matter or ash content. Rather, many steps were conducted to determine other thermal properties of the materials not discussed in the present paper.

The tested samples were weighed inside the crucible and uniformly distributed to avoid internal gradients of heat and gas concentration [4]. However, a temperature gradient inside the particles was not considered due to the small size and quantity of the samples [2,17]. Because the volatile content is strongly affected by the heating rate, the results were not compared to those from previous studies [11,12].

Moisture content was determined by heating the sample to 378 K in an N_2_ atmosphere until a constant weight was achieved. The moisture content (M) was obtained from the following equation: M = 100· (m_1_ – m_2_)/m_1,_ where m_1_ (10^−6^ kg) is the initial mass of the sample and m_2_ the constant mass at 378 K. The volatile matter was determined as the weight loss due to heating from 378 (step 5) to 873 K (step 10) in an N_2_ atmosphere. The volatile content (V) was calculated according to the following equation: V = 100· (m_2_ – m_3_)/m_1_, where m_3_ (10^−6^ kg) is the mass of the sample at 873 K. Ash is the residual inorganic matter remaining after combustion, and the ash content was obtained from the equation A = 100·m_4_/m_1_, where m_4_ (10^−6^ kg) is the mass remaining after step 13. Subsequently, the amount of fixed carbon (FC) was determined from the formula FC = 100 – M – V – A, where A, V and FC were calculated on a dry weight basis (db) and M was calculated on a wet basis (wb).

### 2.4. Statistical treatment

For the determination of the maximum error, the statistical treatment used in this study has been fully described in [11,12,16]. Assuming that the sampling error follows a normal distribution (*SE~N(0,σ(SE*)), as Central Limit Theorem states, we can ensure with a confidence level of 95% that

(1) $$|SE|\leq {SE}_{\text{max}}=1.96\sqrt{\frac{2{HI}_{L}}{n}}$$

and

(2) $$n_{\text{min}}\geq 7.68\frac{{HI}_{L}}{{SE}_{\text{max}}^{2}}$$

where *SE_max_* is the upper bound of the sampling error for a given sampling size (n), n_min_ is the minimum sampling size for a given sampling error and *HI_L_* is the heterogeneity invariant [3,11,12,16].

Because moisture, volatile matter and ash content are measured variables, *SE_max_* represents the maximum sampling error. The amount of fixed carbon (*FC*) was obtained directly from the properties of the materials: $\bar{FC}=(100-\bar{\text{M}}-\bar{\text{V}}-\bar{\text{A}})$. Also, the maximum error was calculated by the method of error propagation, which is fully described in the literature [12]:

(3) $${SE}_{\text{max}}(FC)=\sqrt{\frac{7.68}{M_{m}}\times \frac{{\bar{M}}^{2}{HI}_{L}(M)+{\bar{V}}^{2}{HI}_{L}(V)+{\bar{A}}^{2}{HI}_{L}(A)}{{\left(100-\bar{M}-\bar{V}-\bar{A}\right)}^{2}}}$$

*M̄*, *V̄*, *Ā* and $\bar{FC}$ are the average moisture, volatile matter, ash and fixed carbon content, respectively.

Another objective of this study was the determination of confidence intervals which has been fully described in [3].

## 3. Results and Discussion

Moisture (*wb*), volatile matter (*db*), fixed carbon (*db*) and ash content (*db*) of the samples are presented in Table 3, including the mean and variance of each variable.

*HI_L_*, the heterogeneity invariant, was calculated according to the method described and is summarized in Table 4. The maximum sampling error of a sample with a fixed mass was obtained from the *HI_L_*, and the minimum sample size corresponded to a fixed sampling error. The minimum sample size and maximum sampling error associated with the determination of moisture, volatile matter, fixed carbon and ash content are provided in Tables 5 and 6, 7 and 8, 9 and 10, and 11 and 12, respectively.

To show the utility of the minimum sample mass required to achieve an accurate representation of M, V, A and FC (Tables 5, 7, 9 and 11) and an inverse calculation of the previous one (Tables 6, 8, 10 and 12), examples were performed in [3].

According to the methodology described, confidence intervals of 95% for the properties of each material were generated. Examples for the determination of the confidence intervals were performed in [3]. To compare the results of the present paper to those of previous studies, confidence intervals for the prompt analysis presented in the literature [12] were calculated. The mean weights of the samples in TG analysis were approximately 1000 times less than those of the prompt analysis [12]. Thus, the confidence intervals of TG should be significantly wider ( $\sqrt{1000}=31.623$ times). However, the accuracy of TG equipment compensates for a smaller sample weight, leading to confidence intervals that are approximately five-times greater than those of the prompt analysis. A similar conclusion was achieved in [3].

**Figure 1:**
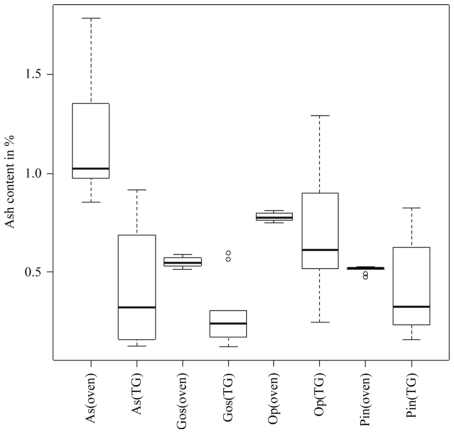
Box-plots of the TG (TG) and prompt (oven) analysis [12] of ash content. Symbol “O”. represents outliers.

Volatile matter and fixed carbon contents obtained from the TG and prompt analysis are not comparable because the results are dependent on the thermal history of the particles, which are completely different in the prompt and TG analysis. However, the moisture content of the materials should be comparable. As shown in Table 13, the mean moisture content obtained in the TG analysis was lower (except Op) than the mean moisture content of the prompt analysis (same conclusion in [3]). Moreover, the mean ash content obtained from TG analysis was lower than the mean ash content of the prompt analysis (same conclusion in [3]). A box-plot of ash content illustrating the median, outliers, smallest and largest observation, and lower and upper quartiles are shown in Figure 1. The results indicated that the ash content obtained from the TG and prompt analyses were not comparable due to the methodology of the TG analysis. The ash content obtained from TG analysis was uniformly lower than that of the prompt analysis. Therefore, biomass heterogeneity was a likely cause for the discrepancy in the results. Due to the low sample weight (20 × 10^−6^ kg), TG crucibles were loaded with tweezers. These favor large particles against small particles and dust that have higher content in ash, as was demonstrated in [3]. It is not possible to assure that the particle size distribution of the materials in the TG analysis is identical to that of the prompt analysis. As such, the mean ash content of these methods is not comparable. A similar explanation is proposed for the determination of moisture content. In general, these results indicate that the mean ash and moisture content obtained from the TG and prompt analysis are not comparable when the proposed methodology is applied. Thus far, all conclusions presented herein are in agreement with those obtained in paper [3].

To study the correlation between properties for the same material, Pearson correlation coefficients were calculated. Moisture, volatile matter and ash content of the materials were considered. Fixed carbon was excluded from this study since it was calculated from the former properties. For a significance level of *α* = 0.05, only ash and moisture content of oak pellets (Op) showed a non-negligible Pearson correlation coefficient of −0.68. Thus, for all other properties and materials, the value of one property cannot be explained from the others because properties are not linearly related. All three variables must be studied separately, and the analysis of one property cannot be used to infer the value of others. Similar conclusions were previously made for prompt analysis [12] and TG [3].

Even though the properties of TG and prompt analysis are not related, the maximum sampling error can be extrapolated from one analysis to the other using equation (1). The maximum sampling error of the materials from the prompt analysis [12] was extrapolated to the TG analysis; the extrapolated error was greater than the maximum sampling error obtained from TG analysis. To illustrate this result, the maximum sampling error of the moisture content of almond shell (As) was extrapolated as an example. According to the literature results [12], *HI_L_*· = 1.55 × 10^−5^ and the maximum sampling error for a sample with an average weight of 23.9 × 10^−3^ kg is 1.09 × 10^−2^. By taking into account the relationship between the average weights of both analyses, the maximum sampling error of TG analysis can be estimated as:

(4) $${\hat{SE}}_{max}(TG)=\sqrt{7.68\cdot 1.55\cdot 10^{-5}\cdot \frac{23.9\cdot 10^{-3}kg}{21.53\cdot 10^{-6}kg}}=0.3635$$

**Figure 2:**
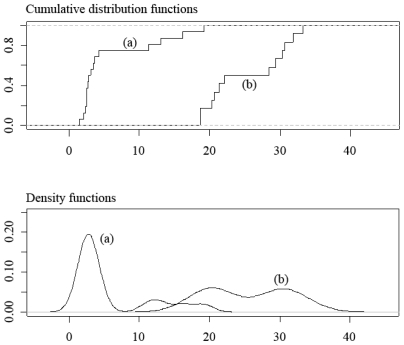
Distribution of the rate of maximum sampling errors for four materials: As, Gos, Op and Pin. (**a**) Cumulative distribution and density functions of *SE_max_*(*TG*)*/SE_max_*(*prompt*) (**b**) Cumulative distribution and density functions of ${\hat{\mathrm{SE}}}_{\max}(\mathrm{TG})/SE_{\max}(\mathrm{prompt})$.

This result does not agree with those shown in Table 6, where *SE_max_(TG)* = 1.23 × 10^−1^. The analysis was repeated for all materials and properties. Values of *SE_max_* (*TG*) */SE_max_* (*prompt*) varied from 1 to 19 while values of ${\hat{SE}}_{max}(TG)/{SE}_{max}(prompt)$ varied from 18 to 33. The cumulative distribution and density functions of both quotients are shown in Figure 2. The results suggested that *SE_max_(TG)* cannot be estimated from *SE_max_(prompt). SE_max_(TG)/SE_max_(prompt)* reached a maximum value of 19 because atypical values were present in the density distribution function (Figure 2 (a)). However, when atypical values were removed, the maximum quotient was equal to 11. The *HI_L_* of the TG and prompt analyses are very different, which explains the lack of relationship between the maximum sampling errors of the methods. As shown previously, the maximum sampling error of the TG analysis should be significantly greater (18–33 times) than that of the prompt analysis. However, the accuracy of TG equipment compensates for the small sample weight, leading to maximum sampling errors that are approximately 1–11 times greater than *SE_max_(prompt).*

**Figure 3:**
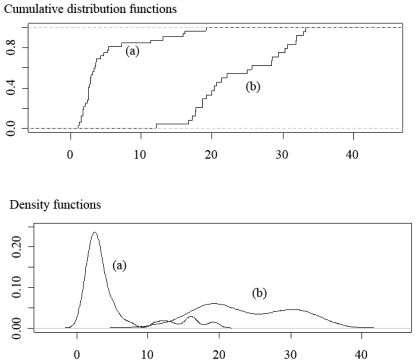
Distribution of the rate of maximum sampling errors for eight materials: Hs, Pns, Pp, Bp, As, Gos, Op and Pin. (**a**) Cumulative distribution and density functions of *SE_max_*(*TG*)*/SE_max_* (*prompt*) (**b**) Cumulative distribution and density functions of ${\hat{\mathrm{SE}}}_{\max}(\mathrm{TG})/SE_{\max}(\mathrm{prompt})$.

Similar results were previously obtained for the same authors and other materials. Figure 3 shows the distribution of the two quotients of maximum sampling errors for eight materials — those studied in paper [3] and those considered in the present work. After consideration of Figures 2 and 3, the conclusion is that independent of the material or the property considered, the maximum sampling error cannot be extrapolated from one analysis to the other.

To observe other relationships between *SE_max_(TG)* and *SE_max_(prompt),* a classical correlation study was conducted on the sampling error *SE_max_(TG)* associated with the volatile matter, fixed carbon and ash content, and the corresponding *SE_max_(prompt)* [12]. A significant correlation coefficient of 0.69 was obtained with p-value of 0.012. Although the correlation is significant, the low value of the correlation coefficient suggests that high levels of error would be encountered if *SE_max_(TG)* was estimated from *SE_max_(prompt)*.

Since measurements of the properties have a natural temporal ordering, some additional analyses were made to check if there was an underlying time series structure. The traditional approach of time analysis is that series consists of three components whose joint action results in the measured values. These components are trend, seasonal variation and random variation. Trend is usually estimated by polynomial regression techniques. Seasonal variation is the periodic oscillations of a short period and is a causal component due to the influence of certain phenomena that occurs periodically. As the sequence of observations is not sufficiently long in time, the seasonal component has not been considered in the present paper. Once this trend has been removed, the residue of the fitted model shows the random variation pattern which, in time series, is correlated in time.

The linear trend and correlation in time of the random variation component were studied for the properties measured in the TG analysis. In total, eight materials were considered — four from paper [3] (hazelnut shell (Hs), pine nut shell (Pns), poplar pellets (Pp) and brassica pellets (Bp)) and four studied in the present work (almond shell (As), ground olive stone (Gos), oak pellets (Op) and pine pellets (Pin)). Table 14 summarizes the results of the statistical analysis applied to the sample data. The first two columns are used to verify the existence of linear trend by means of the Pearson correlation coefficient and the corresponding p-value, respectively. The third and fourth columns show the p-values of the Ljung–Box test, a statistical hypothesis test used to check the null hypothesis that the residues of a time series are not correlated.

For a significance level of *α* = 0.05, only pellets poplar (Pp) has a significant trend for three of its properties: moisture, volatile matter and fixed carbon. Once the trend has been removed, the Ljung-Box test detects correlation in time for several of the properties studied. In the particular cases of moisture of Hs, fixed carbon of Bp and fixed carbon of pine pellets Pin, this correlation remains through two lags in time.

## 4. Conclusions

In this article, statistical analyses of the sampling error and level of uncertainty associated with the properties measured in a TG analysis, as well as the corresponding confidence intervals, were conducted for four types of biomass. Results demonstrated that the sampling procedure and statistical techniques used in this study can be extrapolated to any other solid material in granular form that possesses a homogeneous particle size distribution. Additionally, a study of trends and time correlations was presented for eight types of biomass.

This method is useful for energetic biomass applications where precision has significant importance. Despite the heterogeneity of biofuels, a well planned selection of samples can lead to an extrapolation of sample properties from a large batch. Additionally, the high accuracy of TG equipment compensates for the low sample weight, producing confidence intervals that are smaller than expected.

A comparison between the results obtained with TG and prompt analyses was made. The mean values and maximum sampling errors were not correlated. Additionally, the mean and error of one analysis cannot be used to estimate the mean and error of the other method.

Significant linear trends and correlations in time of the random variation component were detected; however, no satisfactory explanation was found. This must be taken into account in future research.

## Figures and Tables

**Table 1:** Average weights of samples.

Material	Sample Weight (kg)
Almond shell (As)	21.53 × 10^−6^
Ground Olive Stone (Gos)	22.44 × 10^−6^
Oak pellets (Op)	21.44 × 10^−6^
Pine Pellets (Pin)	20.70 × 10^−6^

**Table 2:** Thermal evolution of the samples in TG experiments.

Step	1	2	3	4	5	6	7	8	9	10	11	12	13
**Tstart(K)**	303	343	363	378	378	418	418	773	773	873	873	873	973
**Tend(K)**	343	363	378	378	418	418	773	773	873	873	873	973	973
SR*(K/min)	30	15	2	0	10	0	10	0	20	0	0	20	0
**Time (s)**	80	80	450	1800	240	600	2130	3600	300	600	2400	300	600
**Atmosphere**	N_2_	N_2_	N_2_	N_2_	N_2_	N_2_	N_2_	N_2_	N_2_	N_2_	Air	Air	Air

* Scan Rate.

**Table 3:** The moisture, volatile matter, fixed carbon and ash content of each type of biomass. Except for moisture content, all values are reported on a dry weight basis.

Samples 1 to 6.							
Material	Property	Sample 1	Sample 2	Sample 3	Sample 4	Sample 5	Sample 6
**As**	Moisture	10.597	10.785	10.728	11.040	10.944	11.828
	Volatiles	71.993	74.258	73.627	72.818	73.377	73.957
	Fixed Carbon	27.878	25.582	26.214	26.825	25.708	25.825
	Ash	0.129	0.160	0.159	0.357	0.915	0.218
**Gos**	Moisture	10.718	9.968	10.517	11.014	10.747	10.536
	Volatiles	69.960	69.170	68.573	69.334	69.737	68.712
	Fixed Carbon	29.900	30.541	31.303	30.423	29.957	30.721
	Ash	0.139	0.288	0.125	0.243	0.306	0.567
**Op**	Moisture	8.142	7.616	7.418	8.677	8.007	7.364
	Volatiles	75.276	74.354	75.717	75.405	74.962	74.992
	Fixed Carbon	24.205	25.019	22.991	24.348	24.135	23.971
	Ash	0.519	0.627	1.292	0.248	0.903	1.037
**Pin**	Moisture	7.385	6.794	7.327	6.930	7.054	6.548
	Volatiles	78.067	76.654	77.185	78.080	76.412	78.303
	Fixed Carbon	21.106	23.000	22.583	21.294	23.351	21.387
	Ash	0.827	0.346	0.232	0.626	0.236	0.311

Samples 7 to 10, mean and variance.							
**Material**	**Property**	**Sample 7**	**Sample 8**	**Sample 9**	**Sample 10**	**Mean**	**S^2^**
**As**	Moisture	10.069	10.445	10.315		10.750	0.257
	Volatiles	71.490	73.988	74.530		73.338	1.082
	Fixed Carbon	27.712	25.687	24.780		26.246	1.065
	Ash	0.798	0.324	0.690		0.417	0.092
**Gos**	Moisture	11.021	9.977	11.002		10.611	0.168
	Volatiles	70.739	69.939	69.357		69.502	0.454
	Fixed Carbon	28.665	29.858	30.471		30.204	0.544
	Ash	0.597	0.202	0.173		0.293	0.031
**Op**	Moisture	7.709	7.813	7.799	7.746	7.829	0.145
	Volatiles	74.661	75.481	74.173	76.654	75.167	0.517
	Fixed Carbon	24.738	23.967	25.564	22.622	24.156	0.765
	Ash	0.601	0.552	0.263	0.725	0.677	0.107
**Pin**	Moisture	7.155	7.456	7.333	5.589	6.957	0.314
	Volatiles	76.598	78.070	76.492	76.064	77.193	0.730
	Fixed Carbon	23.204	21.768	23.072	23.212	22.398	0.820
	Ash	0.198	0.162	0.436	0.724	0.410	0.056

**Table 4:** The intrinsic heterogeneity of the moisture, volatile matter, fixed carbon and ash content of different biomass materials.

HI_L_				
	Moisture	Volatiles	Fixed Carbon	Ash
**As**	1.98 × 10^−3^	1.79 × 10^−4^	1.37 × 10^−3^	4.70 × 10^−1^
**Gos**	1.32 × 10^−3^	8.36 × 10^−5^	5.30 × 10^−4^	3.16 × 10^−1^
**Op**	2.12 × 10^−3^	8.24 × 10^−5^	1.18 × 10^−3^	2.11 × 10^−1^
**Pin**	5.83 × 10^−3^	1.10 × 10^−4^	1.47 × 10^−3^	2.99 × 10^−1^

**Table 5:** The minimum sample mass (expressed as n_min_ sampling units) required to achieve a pre-determined maximum sampling error for the determination of moisture content.

		Minimum sample size for a determined sampling error			
		As	Gos	Op	Pin
	***HIL***	1.98 × 10^−3^	1.32 × 10^−3^	2.12 × 10^−3^	5.83 × 10^−3^
**Maximum error**	0.001	1.52 × 10^4^	1.02 × 10^4^	1.63 × 10^4^	4.48 × 10^4^
	0.005	6.07 × 10^2^	4.07 × 10^2^	6.53 × 10^2^	1.79 × 10^3^
	0.01	1.52 × 10^2^	1.02 × 10^2^	1.63 × 10^2^	4.48 × 10^2^
	0.05	6.07	4.07	6.53	17.90

**Table 6:** The maximum sampling error *SE_max_* that corresponds to a given sample mass (expressed as n sampling units) for the determination of moisture content.

		Maximum error for the sample size			
		As	Gos	Op	Pin
	***HIL***	1.98 × 10^−3^	1.32 × 10^−3^	2.12 × 10^−3^	5.83 × 10^−3^
**Sample size**	1	1.23 × 10^−1^	1.01 × 10^−1^	1.28 × 10^−1^	2.12 × 10^−1^
	10	3.90 × 10^−2^	3.19 × 10^−2^	4.04 × 10^−2^	6.70 × 10^−2^
	100	1.23 × 10^−2^	1.01 × 10^−2^	1.28 × 10^−2^	2.12 × 10^−2^
	200	8.71 × 10^−3^	7.13 × 10^−3^	9.03 × 10^−3^	1.50 × 10^−2^

**Table 7:** The minimum sample mass (expressed as n_min_ sampling units) that corresponds to a pre-determined maximum sampling error for the determination of volatile matter content.

		Minimum sample size for a determined sampling error			
		As	Gos	Op	Pin
	***HIL***	1.79 × 10^−4^	8.36 × 10^−5^	8.24 × 10^−5^	1.10 × 10^−4^
**Maximu m error**	0.001	1.37 × 10^3^	6.42 × 10^2^	6.33 × 10^2^	8.48·10^2^
	0.005	54.90	25.70	25.30	33.90
	0.01	13.70	6.42	6.33	8.48
	0.05	5.49 × 10^−1^	2.57 × 10^−1^	2.53 × 10^−1^	3.39 × 10^−1^

**Table 8:** The maximum sampling error, *SE_max_* that corresponds to a given sample mass (expressed as n sampling units) for the determination of volatile matter content.

		Maximum error for the sample size			
		As	Gos	Op	Pin
	***HIL***	1.79 × 10^−4^	8.36 × 10^−5^	8.24 × 10^−5^	1.10 × 10^−4^
**Sample size**	1	3.71 × 10^−2^	2.53 × 10^−2^	2.52 × 10^−2^	2.91 × 10^−2^
	10	1.17 × 10^−2^	8.01 × 10^−3^	7.96 × 10^−3^	9.21 × 10^−3^
	100	3.71 × 10^−3^	2.53 × 10^−3^	2.52 × 10^−3^	2.91 × 10^−3^
	200	2.62 × 10^−3^	1.79 × 10^−3^	1.78 × 10^−3^	2.06 × 10^−3^

**Table 9:** The minimum sample mass required for the determination of fixed carbon content (expressed as n_min_ sampling units) for a pre-determined maximum sampling error.

		Minimum sample size for a determined sampling error			
		As	Gos	Op	Pin
	***HIL***	1.37 × 10^−3^	5.30 × 10^−4^	1.18 × 10^−3^	1.47 × 10^−3^
**Maximum error**	0.001	1.06 × 10^4^	4.07 × 10^3^	9.07 × 10^3^	1.13 × 10^4^
	0.005	4.22 × 10^2^	1.63 × 10^2^	3.63 × 10^2^	4.52 × 10^2^
	0.01	1.06 × 10^2^	40.70	90.70	1.13 × 10^2^
	0.05	4.22	1.63	3.63	4.52

**Table 10:** The maximum sampling error, *SE_max_* that corresponds to a given sample mass (expressed as n sampling units) for the determination of fixed carbon content.

		Maximum error for the sample size			
		As	Gos	Op	Pin
	***HIL***	1.37 × 10^−3^	5.30 × 10^−4^	1.18 × 10^−3^	1.47 × 10^−3^
**Sample size**	1	1.03 × 10^−1^	6.38 × 10^−2^	9.52 × 10^−2^	1.06 × 10^−1^
	10	3.25 × 10^−2^	2.02 × 10^−2^	3.01 × 10^−2^	3.36 × 10^−2^
	100	1.03 × 10^−2^	6.38 × 10^−3^	9.52 × 10^−3^	1.06 × 10^−2^
	200	7.27 × 10^−3^	4.51 × 10^−3^	6.73 × 10^−3^	7.52 × 10^−3^

**Table 11:** The minimum sample mass required for the determination of ash content (expressed as n_min_ sampling units) for a pre-determined maximum sampling error.

		Minimum sample size for a determined sampling error			
		As	Gos	Op	Pin
	***HIL***	4.70 × 10^−1^	3.16 × 10^−1^	2.11 × 10^−1^	2.99 × 10^−1^
**Maximum error**	0.001	3.61 × 10^6^	2.43 × 10^6^	1.62 × 10^6^	2.30 × 10^6^
	0.005	1.45 × 10^5^	9.71 × 10^4^	6.48 × 10^4^	9.18 × 10^4^
	0.01	3.61 × 10^4^	2.43 × 10^4^	1.62 × 10^4^	2.30 × 10^4^
	0.05	1.45·10^3^	9.71·10^2^	6.48·10^2^	9.18·10^2^

**Table 12:** The maximum sampling error, *SE_max_* that corresponds to a given sample mass (expressed as n sampling units) for the determination of ash content.

		Maximum error for the sample size			
		As	Gos	Op	Pin
	***HIL***	4.70 × 10^−1^	3.16 × 10^−1^	2.11 × 10^−1^	2.99 × 10^−1^
**Sample size**	1	1.90	1.56	1.27	1.52
	10	6.01 × 10^−1^	4.93 × 10^−1^	4.03 × 10^−1^	4.79 × 10^−1^
	100	1.90 × 10^−1^	1.56 × 10^−1^	1.27 × 10^−1^	1.52 × 10^−1^
	200	1.34 × 10^−1^	1.10 × 10^−1^	9.00 × 10^−2^	1.07 × 10^−1^

**Table 13:** Confidence intervals of 95% for the TG and prompt analysis of moisture (wb), volatile matter (db), fixed carbon (db) and ash (db) content [12].

		Moisture	Volatiles	Fixed Carbon	Ash
**As**	TG	10.75 ± 4.42 × 10^−1^	73.34 ± 9.06 × 10^−1^	26.25 ± 8.99 × 10^−1^	0.42 ± 2.64 × 10^−1^
	Prompt	12.59 ± 4.58 × 10^−2^	78.38 ± 3.55 × 10^−1^	20.44 ± 2.83 × 10^−1^	1.17 ± 2.65 × 10^−1^
**Gos**	TG	10.61 ± 3.57 × 10^−1^	69.50 ± 5.87 × 10^−1^	30.20 ± 6.42 × 10^−1^	0.29 ± 1.52 × 10^−1^
	Prompt	12.62 ± 1.23·10^−1^	79.83 ± 2.84 × 10^−1^	19.62 ± 2.52 × 10^−1^	0.55 ± 2.19 × 10^−2^
**Op**	TG	7.83 ± 3.16 × 10^−1^	75.17 ± 5.98 × 10^−1^	24.16 ± 7.27 × 10^−1^	0.68 ± 2.72 × 10^−1^
	Prompt	7.51 ± 1.45 × 10^−1^	79.07 ± 2.63 × 10^−1^	20.15 ± 2.54 × 10^−1^	0.78 ± 1.64 × 10^−2^
**Pin**	TG	6.96 ± 4.66 × 10^−1^	77.19 ± 7.11 × 10^−1^	22.40 ± 7.53 × 10^−1^	0.41 ± 1.96 × 10^−1^
	Prompt	7.38 ± 1.59 × 10^−1^	80.60 ± 1.75 × 10^−1^	18.88 ± 1.74 × 10^−1^	0.52 ± 1.53 × 10^−2^

**Table 14:** Study of the linear trend and the random variation components for the properties of eight materials.

		Pearson correlation		Ljung-Box test	
		
		coefficient	p-value	p-value for 1 lag	p-value for 2 lags
**Gos**	Moisture	0.189	0.626	0.126	0.302
	Volatiles	0.244	0.527	0.364	0.346
	Fixed Carbon	−0.294	0.443	0.504	0.399
	Ash	0.298	0.436	0.400	0.234
	
**As**	Moisture	−0.241	0.532	0.666	0.890
	Volatiles	0.272	0.478	0.327	0.281
	Fixed Carbon	−0.446	0.229	0.458	0.172
	Ash	0.584	0.099	0.057	0.088
	
**Hs**	Moisture	0.337	0.375	**0.032**	**0.022**
	Volatiles	0.104	0.790	0.743	0.104
	Fixed Carbon	−0.154	0.693	0.829	0.101
	Ash	0.516	0.155	**0.251**	0.466
	
**Bp**	Moisture	0.653	0.232	0.234	0.222
	Volatiles	−0.491	0.401	0.089	0.147
	Fixed Carbon	0.372	0.538	**0.041**	**0.054**
	Ash	0.623	0.262	0.957	0.085
	
**Pp**	Moisture	**0.909**	**0.033**	0.916	0.141
	Volatiles	**0.995**	**0.000**	0.150	0.300
	Fixed Carbon	**−0.990**	**0.001**	0.465	0.205
	Ash	0.043	0.945	0.370	0.327
	
**Pin**	Moisture	−0.379	0.280	0.623	0.544
	Volatiles	−0.371	0.291	**0.048**	0.091
	Fixed Carbon	0.388	0.268	**0.015**	**0.029**
	Ash	−0.144	0.691	0.965	0.662
	
**Pns**	Moisture	−0.559	0.118	0.835	0.905
	Volatiles	−0.509	0.161	0.452	0.607
	Fixed Carbon	0.362	0.338	0.847	0.835
	Ash	0.145	0.710	0.957	0.802
	
**Op**	Moisture	−0.186	0.607	0.549	**0.034**
	Volatiles	0.198	0.584	0.078	0.165
	Fixed Carbon	−0.096	0.793	**0.049**	0.109
	Ash	−0.180	0.620	0.171	0.310
